# Human Galectin-7 Gene *LGALS7* Promoter Sequence Polymorphisms and Risk of Spontaneous Intracerebral Hemorrhage: A Prospective Study

**DOI:** 10.3389/fnmol.2022.840340

**Published:** 2022-03-23

**Authors:** Ming-Dong Wang, Jing Tian, John H. Zhang, Shun-Ying Zhao, Ming-Jing Song, Zhan-Xiang Wang

**Affiliations:** ^1^Department of Neurosurgery, The First Affiliated Hospital of Xiamen University, School of Medicine, Xiamen University, Xiamen, China; ^2^Beijing Children’s Hospital, Capital Medical University, National Center for Children’s Health, China National Clinical Research Center of Respiratory Disease, Beijing, China; ^3^Physiology Program, Department of Anesthesiology, Neurosurgery, Neurology, and Physiology, Center for Neuroscience Research, Loma Linda University School of Medicine, Loma Linda, CA, United States; ^4^Medical School, Huanghe Science and Technology University, Zhengzhou, China; ^5^Institute of Laboratory Animal Science, Chinese Academy of Medical Sciences and Comparative Medicine Center, Peking Union Medical College, Beijing, China

**Keywords:** cerebral hemorrhage, galectin-7, promoter, single-nucleotide polymorphism, genetic

## Abstract

**Background and purpose:**

Despite evidence for the role of genetic factors in stroke, only a small proportion of strokes have been clearly attributed to monogenic factors, due to phenotypic heterogeneity. The goal of this study was to determine whether a significant relationship exists between human galectin-7 gene *LGALS7* promoter region polymorphisms and the risk of stroke due to non-traumatic intracerebral hemorrhage (ICH).

**Methods:**

This two-stage genetic association study included an initial exploratory stage followed by a discovery stage. During the exploratory stage, transgenic galectin-7 mice or transgenic mice with the scrambled sequence of the hairpin structure –silenced down gene *LGALS7*—were generated and then expressed differentially expressed proteins and galectin-7-interacting proteins were identified through proteomic analysis. During the discovery stage, a single-nucleotide polymorphism (SNP) genotyping approach was used to determine associations between 2 *LGALS7* SNPs and ICH stroke risk for a cohort of 24 patients with stroke of the Chinese Han population and 70 controls.

**Results:**

During the exploratory phase, *LGALS7* expression was found to be decreased in TG^LGALS–DOWN^ mice as compared to its expression in TG^LGALS^ mice. During the discovery phase, analysis of *LGALS7* sequences of 24 non-traumatic ICH cases and 70 controls led to the identification of 2 ICH susceptibility loci: a genomic region on 19q13.2 containing two *LGALS7* SNPs, rs567785577 and rs138945880, whereby the A allele of rs567785577 and the T allele of rs138945880 were associated with greater risk of contracting ICH [for T and A vs. C and G, unadjusted odds ratio (OR) = 13.5; 95% CI = 2.249–146.5; *p* = 0.002]. This is the first study to genotype the galectin-7 promoter in patients with hemorrhagic stroke. Genotype and allele association tests and preliminary analysis of patients with stroke revealed that a single locus may be a genetic risk factor for hemorrhagic stroke.

**Conclusion:**

A and T alleles of two novel SNP loci of 19q13.2, rs567785577 and rs138945880, respectively, were evaluated for associations with susceptibility to ICH. Further studies with expanded case numbers that include subjects of other ethnic populations are needed to elucidate mechanisms underlying associations between these SNPs and ICH risk.

## Introduction

Stroke is a multifactorial disease that leads to age-related disability, cognitive decline, and dementia. Stroke risk reflects the presence of modifiable clinical risk factors that include blood pressure, diabetes, lipid metabolism disorders, atrial fibrillation, and coronary artery disease (Wolf et al., 1991; Apostolis et al., 2014), and also genetic risk factors, as revealed in family studies. In general, most common human diseases appear to be associated with rare mutations of certain genes that confer to heterozygous carriers several-fold increased risk of disease (Amit V Khera et al., 2018). Notably, the heritability of spontaneous non-traumatic intracerebral hemorrhage (ICH) phenotype has been estimated to be as high as 44% (Carpenter et al., 2016). Due to the fact that ICH has 30-day mortality of approximately 40% (Qureshi et al., 2005), stroke is viewed as a devastating disease, which prompts researchers to strive to identify ICH risk factors, of which an estimated 30% have yet to be identified. Meanwhile, studies have found that roughly 8% of the population have inherited a genetic predisposition that confers a threefold increased risk for coronary artery disease (Carpenter et al., 2016). In addition, having a first-degree relative who experienced an ICH stroke event increases an individual’s odds of developing ICH by sixfold (Woo et al., 2002). Thus, genome research is a powerful tool for enhancing our understanding of how genetic variation impacts stroke risk that should eventually help clinicians prevent a stroke from occurring in high-risk individuals.

Risk factors for stroke and associated disorders are known to include environmental and genetic factors, although associations between phenotypic disease manifestations and genes are still unclear, but are of special interest to neurological clinicians. During the past few years, numerous reports of new gene loci associated with increased ICH stroke susceptibility and increased risks of stroke-associated clinical complications, such as increased hematoma volume, poor prognosis, stroke recurrence, and mortality, have been reported. Such loci include *APOE*, *COL4A1*, *COL4A2*, *CD36*, *TIMP-1*, *TIMP-2*, *MMP-2*, *MMP-9*, *KCNK17*, *CR1*, *STYK1*, *ACE*, *1Q22*, and *CETP* (Slowik et al., 2004; Chen et al., 2008; Kalita et al., 2011; Woo et al., 2014; Ho et al., 2015; Anderson et al., 2016; Gong et al., 2017; Rannikmae et al., 2017; Lin et al., 2018; Sawyer et al., 2018; Marini et al., 2019). Nevertheless, although individual ICH stroke-associated genes follow the Mendelian model of inheritance, ICH stroke is a polygenic disease (Wahab et al., 2019). In addition, an association has been reported between galectins-1, -3, -9, and -12 that are encoded by *LGALS1, 3, 9*, and 12, respectively, that confer greater cardiovascular accident risk to humans (Abel et al., 2019).

Galectins, lectin molecules that bind to β-galactosides, belong to the LGALS family of proteins, a family that includes three distinct groups of molecules (prototypic, chimeric, tandem-repeat) that bind to carbohydrate molecules *via* carbohydrate recognition domains (CRDs). In recent years, the possible roles of galectins in human disease have attracted attention from researchers studying heart disease, atherosclerosis, stroke, and cerebral ischemia (including intracranial and subarachnoid hemorrhage). Nevertheless, in spite of the fact that galectins have been shown to play key roles in human diseases, research studies have been done to determine whether they are the risk factors for non-traumatic intracerebral ischemic hemorrhage (ICH), a common disease afflicting millions of people worldwide. Galectin-7 belongs to the prototypic LGALS group, based on the criteria that each galectin-7 molecule contains one CRD and that galectin-7 molecules can form homodimers upon binding to glycoconjugates (Chiariotti et al., 2002). In contrast to other galectins (e.g., Gal-1 and Gal-3) that are present throughout the human body, galectin-7 appears to be distributed in the mammalian body in tissue- and cell-specific ways that include brain tissues and are involved in various physiological and pathological events (Storan et al., 2004; Nio-Kobayashi et al., 2009). Therefore, due to its tissue distribution, galectin-7 may be associated with stroke, although the association between galectin-7 and stroke risk has not been reported. Here, galectin-7 is assessed as a novel genetic risk factor associated with poor clinical ICH outcomes in patients with stroke of the Chinese Han population.

## Materials and Methods

### Study Design and Patient Population

This exploratory prospective cohort-based, multistage genetic association study was approved by the Ethics Committee of The First Affiliated Hospital of Xiamen University. Written informed consent was obtained from each participant before initial data collection was conducted. Consecutive patients seeking care for a first stroke due to non-traumatic ICH who were admitted to the stroke center within 24 h of symptoms onset between 2012 and 2013 were enrolled after screening to ensure that they fit all study criteria. Ultimately, the final study group consisted of 24 Chinese Han subjects (8 men and 16 women) who had experienced non-traumatic ICH stroke who were in the median age of 49.08 ± 1.86 years. All patients with stroke were recruited from the same stroke center and were identified through surveillance conducted by the staff of hospital emergency and radiology departments based on diagnosis at the time of hospital discharge. Exclusion criteria included ICH caused by vascular structural abnormalities, congenital malformations, tumors, and trauma. All patients were required to consent to genetic testing before they were enrolled in the study. Demographic, clinical, and genotype data of the patient population are shown in Table 1.

**TABLE 1:** Clinical characteristics of study participants.

No. of subjects	Intracerebral hemorrhage (*n* = 24)	Controls group (*n* = 70)	*p*-value
**Sex %** Female Male	13 (54.16%) 11 (45.83%)	35 (50%) 35 (50%)	0.1573
Age (years)	49.08 ± 1.86	37.53 ± 1.47	<0.0001
**History**			
Hypertension %	16/24 (66.67%)	0/70	<0.0001
Diabetes %	8/24 (33.33%)	0/70	<0.0001
Lipid disorders %	16/24 (66.67%)	0/70	<0.0001
**Smoking**			
Never	0/24 (%)	24/70 (34.29%)	0.0004
Former %	4/24 (16.67%)	6/70 (8.75%)	0.3268
Active smoker %	18/24 (75%)	40/70 (57.14%)	0.4611
**Alcohol consumption**			
Never	0/24 (%)	28/70 (40%)	0.0029
Former %	9/24 (14.28%)	10/70 (37.5%)	0.164
Active %	13/24 (45.71%)	32/70 (54.17%)	0.6752

All 70 control group subjects (35 men and 35 women) were neurologically healthy Chinese Han volunteers in the median age of 37.53 ± 1.47 years without histories of stroke, hypertension, diabetes, lipid metabolism, atrial fibrillation, coronary artery disease, Alzheimer’s disease, brain aneurysm, tumor, dementia, dystonia, or Parkinson’s disease. All control subjects provided written consent before undergoing genetic analysis.

### Procedures for Animal Care and Animal Experiments

Animal procedure protocols were approved by the Animal Care and Use Committee of the Institutional of Laboratory Animal Science, Chinese Academy of Medical Sciences in Beijing, China (ILAS-PG-2014-010). Animal experiments were conducted in accordance with the principles outlined in the National Institutes of Health Guide for the Care and Use of Laboratory Animals. The study design was approved by the ethics committee of First Affiliated Hospital of Xiamen University.

In total, 15 groups of 1-month-old mice were used in the study. After the mice were euthanized, blood, vascular, and lung tissues were harvested, and then, tissues were analyzed *via* western blotting (WB), immunohistochemical analysis, and proteomic analysis based on isobaric tags for relative and absolute quantification (iTRAQ) methods. Wild-type (WT) BALB/c mice were obtained from the Institute of Laboratory Animal Science, Chinese Academy of Medical Sciences (Beijing). Mice were housed in cages under temperature-controlled conditions (20–25°C) and a 12-h light–12-h dark cycle. Genotypes of transgenic (TG) and non-TG mice were confirmed by polymerase chain reaction (PCR) amplification of DNA obtained from tail snips. Age-matched WT mice served as controls.

### Cell Culture and Construction of pcDNA3.1-LGALS7, (TG^LGALS^) pSilencer-LGALS7-Recombinant Plasmid Vectors (TG^LGALS–DOWN^), and pGL-3-LGALS7 Promoter

Human embryonic kidney (HEK) 293T cells were grown in Dulbecco’s modified Eagle’s medium (DMEM) (HyClone) supplemented with 10% fetal bovine serum (FBS) (HyClone) and antibiotics (50 mg/ml streptomycin and 50 U/ml penicillin). Cell cultures were maintained at 37°C in a humidified atmosphere containing 5% CO_2_ in a tissue culture incubator (SHELLAB). Three vectors were used in this study: pGL-3-LGALS7 promoter, pSilencer™–5.1-U6Retro, and pcDNA3.1 (please refer to Supplementary Appendix 1, vector design and construction). PCR was carried out using respective gene-specific primers (Table 2).

**TABLE 2:** Sequences of primers used in this study.

	Upstream stem (_sequence from 5’to 3’_)	Downstream stem (_sequence from 5’to 3’_)	Products
Transgenic mice	GCCCGCCATGTCTGCTACCC	CGTCATCCCCGACCACAGCC	323 bp
Transgenic mice	TACCCAGCACAAGACCTCCCT	TCTCCGCCCACCTCCACCAGC	372 bp
Interfere with mice	ATCCTCCCTTTATCCAGC	GAGGGCCTATTTCCCATG	392 bp

The DNA region that contains the human galectin-7 gene (*LGALS7*) promoter region was obtained through *Kpn*I and *Xho*I digestion of T-vector pMD19. This DNA fragment was then purified and ligated to pGL-3 to generate the plasmid pGL-3-LGALS7 promoter, a plasmid containing *LGALS7* promoter sequence DNA inserted upstream and in frame with dual-luciferase reporter coding sequences. The pSilencer-LGALS7-Recombinant Plasmid Vector DNA construct contained a silencing hairpin RNA (shRNA) sequence inserted between two restriction sites of pSilencer™-5.1-U6Retro vector (Ambion); when the shRNA was transcribed, it suppressed the generation of *LGALS* mRNA transcripts, thereby silencing galectin-7 expression. Meanwhile, the 3.7-kb human surfactant protein C (SPC) insert was excised from the 3.7SPC/SV40 vector through *Nde*I, and *Eco*RI digestion then was cloned into pcDNA3.1(+). Next, galectin-7-encoding cDNA was inserted into this vector using two other restriction sites to generate pcDNA3.1-LGALS7 recombinant plasmid.

### Microinjection of LGALS Constructs to Generate Transgenic (TG) Mice Expressing LGALS7 and TG Mice With Silenced LGALS7 Expression Resulting From RNA Interference-Mediated Gene Knockdown

TG^LGALS^ and TG^LGALS–DOWN^ (shRNA) mice were generated by microinjection of pcDNA3.1-LGALS7 and pSilencer-LGALS7 plasmid vectors into fertilized oocytes. Approximately 1–2 nl of each DNA construct was injected into one-cell-stage fertilized eggs to generate separate transgenic lines that were maintained in our animal facility by backcrossing them with BALB/c mice. Genotypes of transgenic founders and offspring were identified using PCR amplification of tail snip DNA of TG mice using transgene-specific primers (Tian et al., 2021).

The full-length *LGALS7* cDNA sequence was reverse-transcribed from *LGALS7* mRNA followed by PCR amplification of the DNA using primers derived from the *LGALS7* sequence. After confirmation of PCR product size and quality *via* agarose gel electrophoresis, the PCR product was cloned into the pcDNA3.1 vector. The cloned PCR product was confirmed to be the galectin-7-coding sequence of the mouse *LGALS7* gene *via* DNA sequencing prior to the generation of TG mice overexpressing galectin-7. To confirm that TG mice were generated correctly, DNA was extracted from tail snips, and then, genotyping was performed *via* PCR using primers specific for each transgene (Table 2).

### Luciferase Reporter Gene Assays

**FIGURE 1:**
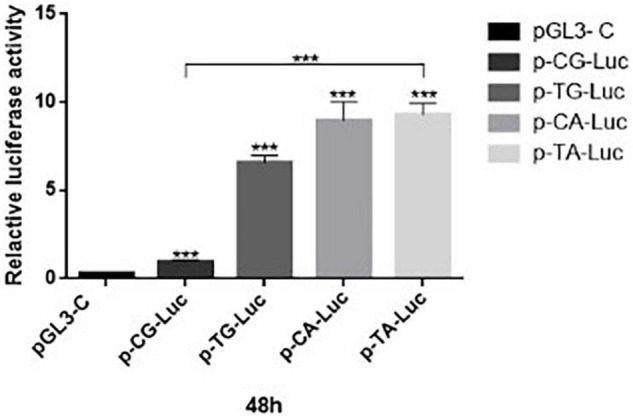
Detection of dual-luciferase activity of 293T cells at 48 h after transfection. Promoter activities of recombinant plasmids p-CG-Luc (*p* = 0.003, *t* = 4.88), p-TG-Luc (*p* < 0.05, *t* = 16.38), p-CA-Luc (*p* < 0.05, *t* = 8.19), and p-TA-Luc (*p* < 0.05, *t* = 39.34), p-CG-Luc, p-TG-Luc, p-CA-Luc, and p-TA-Luc were much higher than that of the negative control plasmid PGL3-control with SV40 promoter. ***P < 0.001 three asterisks signify less than 0.001, indicating a significant difference, statistically significant.

HEK 293T cells were cultured and propagated according to the growth conditions described above. Dual-luciferase reporter assays were conducted when cells reached 70-80% confluence for two experimental groups and one control group (control of plasmid transfection efficiency refer to Supplementary Appendix 1, page 5). Cells in both experimental groups received reporter constructs and substrates that were mixed together then adjusted with serum-free diluent to a total volume of 25 μl that included 0.4 μg of pGL3-Basic-promoter with C and G, and 0.02 μg of pRL-TK in one experimental group or 0.4 μg of pGL3-Basic-T and A, and 0.02 μg of pRL-TK in the other experimental groups or 0.4 μg of pGL3-Basic-T and G, and 0.02 μg of pRL-TK in the other experimental groups or 0.4 μg of pGL3-Basic-C and A, and 0.02 μg of pRL-TK in other experimental group, cells in the control group received 0.4 μg of pGL3-control and 0.02 μg of pRL-TK. Thereafter, recombinant plasmid reporter construct DNAs and transfection solution (Lip2000Neofect™ 1 μl) were added to cells of all groups after adjustment of total volumes to 25 μl by the addition of serum-free diluent. After 48 h of culture, the cells were collected from wells of the 96-well plates, and then, promoter activity based on luciferase activity was detected using a multifunction microplate reader (Figure 1).

### Western Blot Analysis

After proteins were identified *via* mass spectrometry, their identities were verified using WB analysis (refer to Supplementary Appendix 1). Total protein of TG mice was extracted from pulmonary vascular tissues, and then, protein concentrations were determined manually based on mass balance calculations and then were verified based on absorbances that were determined using a benchtop UV (ultraviolet) spectrophotometer. Next, protein samples were loaded onto 10% polyacrylamide gels, and then, proteins were separated *via* SDS-PAGE. Thereafter, separated proteins were electroblotted onto polyvinylidene fluoride (PVDF) membranes. Next, membranes were blocked in 5% non-fat milk for 1 h followed by incubation with primary antibodies, washed, and then incubated with secondary antibodies. The following primary antibodies were used: antigalectin-7 (R&D; 1:200 dilution), antitroponin C (ABclonal; 1:200 dilution), antimyosin light chain 3 (ABclonal; 1:200 dilution), antikeratin 13 (ABclonal; 1:200 dilution), antialpha-1 acid glycoprotein 2 (Sigma; 1:200 dilution), and antiactin-1 (Abcam; 1:2,000 dilution). Immunoreactive bands were visualized using enhanced chemiluminescent (ECL) reagent, and then, the optical density of each band was determined using Image Lab 4.1 software.

### Isobaric Tags for Relative and Absolute Quantification

Experimental (TG mice) and control group samples were subjected to proteomic analysis after they were extracted, quantified, proteolytically digested, desalted, and then labeled according to the standard iTRAQ protocols (Thermo Q-Exactive). Refer to Supplementary Appendix 1 iTRAQ. Labeled peptides were next separated *via* high-performance liquid chromatography (HPLC) (two analytical runs/sample). Next, HPLC-separated iTRAQ-labeled peptides were subjected to liquid chromatography-tandem mass spectrometry (LC-MS/MS) analysis using an EASY-nLCTM 1200 UHPLC system (Thermo Fisher) equipped with a Q Exactive HF-X spectrometer operating in the data-dependent acquisition mode. Proteins were identified based on the results of peptide sequence comparisons against the UNIPROT *Mus musculus* database. The original mass spectrometric file was processed using Proteome Discoverer 1.3, commercial software supported by Thermo. After filtering the data to remove proteins associated with different multiples of ≥ 1.2 or ≤ 0.833, the remaining data corresponding to peptides of differentially expressed proteins were subjected to further analysis.

### Genotyping-Based Identification of Galectin-7 Polymorphisms

Research staff who performed genotyping were blinded to clinical outcomes data and clinical diagnosis information. Genotyping of duplicate samples was performed using 20 ng of genomic DNA in a 25-μl reaction volume using TaKaRa Taq™ (TaKaRa, # R001B). Reactions were run using a PCR instrument (Applied Biosystems). During the discovery stage, single-nucleotide polymorphisms (SNPs) were genotyped for the experimental group and controls using Sanger DNA sequencing technology. Next, SNPs were analyzed using Chromas software to determine mutation frequencies and the allele frequency spectrum, and then, *LGALS7* promotor sequence mutations were grouped according to the type of mutation. During the validation stage, significant SNPs identified during the discovery stage were genotyped using Sanger sequencing for a cohort of 24 cases (stroke) and 70 controls (normal volunteers) to verify genotypes and mutations identified during the discovery stage.

### Bioinformatic Analysis

All proteins verified as differentially expressed proteins were grouped based on protein expression profiles determined *via* hierarchical cluster analysis using the Cluster 3.0 program. Next, identified proteins were classified and grouped into different biochemical pathways using the Kyoto Encyclopedia of Genes and Genomes (KEGG) database^1^ and Reactome pathway database (version 70, released on September 9, 2019). The STRING 10 database^2^ was also used to detect functional interactions between identified proteins. To predict the functions of differentially expressed proteins, the proteins were analyzed using gene ontology (GO) analysis, and then, each was assigned to one GO category (biological process, cellular component, or molecular function).

### Clinical Subjects, Genomic DNA Extraction, and Genotyping

A total of 24 clinically diagnosed spontaneous patients with ICH and 70 healthy control participants were recruited by the Stroke Center of Xiamen University. Peripheral whole blood samples were collected in 5 ml tubes containing either sodium heparin or sodium EDTA anticoagulants, and then, tubes were stored at 4°C. To facilitate fast, effective genomic DNA extraction from the 94 samples from patients with ICH and controls, all samples were processed according to the protocol described in the AxyPrep Blood Genomic DNA Miniprep Kit (Axygen). The resulting genomic DNA extracts were quantified *via* spectrophotometry and then were stored at –20°C.

Based on the human *LGALS7* DNA sequence obtained from GenBank (Gene ID: 3963), Primer Premier 5.0 software was used to design PCR primers (as shown in Table 2). Amplifications were performed under the following conditions: predenaturation at 94°C for 5 min followed by 35 cycles of (initial denaturation at 95°C for 45 s, annealing at 55°C for 45 s, and extension at 72°C for 60 s, followed by a final extension step of 72°C). For the complete digestion of PCR fragments, 5 μl of the PCR product was incubated at 65°C for 3 h.

### Statistical Analysis

Statistical analysis of genotyping data was conducted using SNPGWA software. The *t-*test was used to compare the measurement data, and allele frequencies and genotype frequencies were calculated through direct counting of gene alleles. Statistical data were analyzed by univariate test, chi-square test, or Hardy–Weinberg equilibrium (HWE) test, with each result expressed as an odds ratio (OR) and 95% CI. Results that satisfy 95% CI and *p* < 0.05 criteria were deemed statistically significant. Each SNP was tested for departure from HWE expectations based on the study and control group comparison results obtained using the Student’s *t*-test. To identify an association between an individual polymorphism and stroke status, several tests were done using SNPGWA. A statistical significance threshold based on a Bonferroni corrected *p*-value of < 0.025 (*p*-value/number of SNPs tested) was set for the use in interpreting the statistical significance of allele, genotype, and haplotype frequencies between controls and stroke cases. Effects of haplotype or haplotype features on disease risk were calculated using an expectation–conditional–maximization (ECM) algorithm and tools provided with CHAPLIN software.

Different genetic models, such as dominant (MM vs. Mm + mm), recessive (mm vs. Mm + MM), overdominant (MM + mm vs. Mm), and codominant (MM vs. Mm vs. mm), and additive and multiplicative models were checked, and then, the most significant models were applied. The significance of the difference between two independent proportions was calculated using a Z-test with two-tailed probability. Continuous variables are presented as mean ± SE and *p*-values of < 0.05 were considered statistically significant.

## Results

### Identification and Characterization of the Human *LGALS7* Gene in Transgenic Mice

The *LGALS7* gene is located on chromosome 19q13.2, and the promoter sequence containing the two SNPs, rs567785577 and rs138945880, discussed in this work is obtained immediately before the gene start code (please refer to Supplementary Appendix 1, locus Mappping). The full-length cDNA of *LGALS7* is predicted to encode the galectin-7 protein, which is comprised of 136 amino acids.

### Characterization of *LGALS7* Gene Expression in Mice Embryos

**FIGURE 2:**
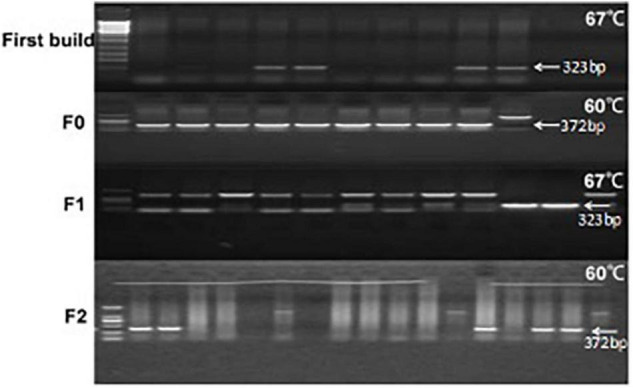
Agarose electrophoresis analysis of PCR amplification products of the galectin-7-encoding gene in the mouse of different generations of TG mice at different annealing temperatures. Each lane was loaded with 5 μl of PCR products. This is a combination diagram of agarose gel electrophoresis reactions of different individual mice, where F0, F1, and F2 represent the algebra of different mice. In the agarose gel electrophoresis figure, from left to right, the first lane on the left is a marker (DL2000-DNA-marker, with sizes of 100, 250, 500, 750, 1,000, and 2,000 bp, respectively. Second, each lane represents an individual. First built mice 12 mice, F0 has 12 mice, F1 has 10 mice, and F2 had 15 mice. Each individual uses two sets of different primers for PCR amplification, the product sizes are 323 bp and 372 bp, respectively, and the reaction system was 25 μl as described in the method. When the first band is unclear or the band is too shallow, it uses the second set of primers to detect coincidence. The positive control is the galactin-7 template (cDNA sequence) ligated in the T-vector—the positive control. A number of 4 individuals were tested two times in the F0 generation.

**FIGURE 3:**
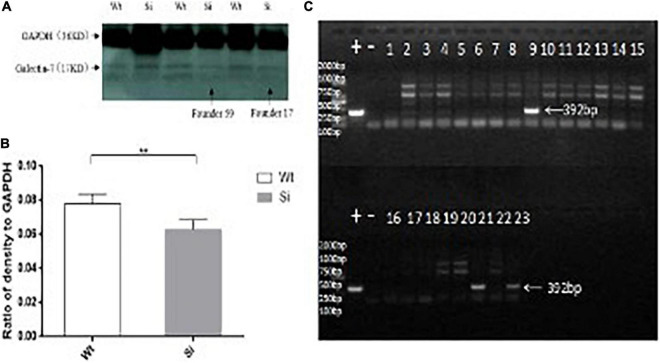
Differential expression of the galectin-7 protein in tissues of TG mice. Panel **(A)** shows transgene expression at the protein level [Tg (+) positive mice in the same litter, Tg (−) negative controls in the same litter]. WB bands are clearly visible in TG mouse samples (Tg +) as 17 kD protein bands, with expression levels presented as means ± SD. *p < 0.01 vs. that of the Tg(–) group. Panel **(B)** shows the expression of galectin-7 protein expression (as determined *via* WB) in knockdown mice and wild-type BALB/c mice, respectively. WT (BALB/c mice), Si (galectin-7 knockdown BALB/c mice). Protein bands with expression levels presented as means ± SD. *p < 0.05 vs. WT group. Panel **(C)** shows a positive control PCR experiment diagram of the interference sequence, and 392 bp is the product of the interfering sequence and interfering sequence vector amplification. *P < 0.05, **P < 0.01, ***P < 0.001. One asterisks signify less than 0.05, indicating a difference, statistically significant.

The annealing temperature expression of *LGALS7* genes in mice was determined by PCR (Figure 2). The results showed that the *LGALS7* gene was overexpressed in tissues of transgenic mice (Figure 3A).

### Knockdown of *LGALS7* Transcription Suppressed Galectin-7 Expression

To determine whether knockdown of *LGALS7* expression led to altered expression of connexins and other proteins that interact with the galectin-7 protein, we first verified that knockdown had been successfully achieved by analyzing gene fragments from galectin-7 knockdown mice *via* PCR (Figure 3C). We then confirmed that galectin-7 protein was reduced in knockdown mice by WB analysis (Figure 3B).

### Analysis of Mice With Overexpressed vs. Silenced *LGALS7*

Based on significant *LGALS7* expression differences observed between TG mice with expressed vs. silenced *LGALS7*, we next focused on sequencing of peptides derived from differentially expressed proteins associated with genetically induced changes in quantitative proteomic profiles and protein interactions. To maximize the chance of successful identification of relevant proteins, HPLC separation of peptides was conducted using a sandwich-based method with gradient elution, with samples processed at a minimum in triplicate with a requirement that peptides be identified two times before they were included in the analysis. Ultimately, MS analysis of labeled iTRAQ peptides led to the identification of a total of 1,009 differentially expressed proteins. After pairwise comparisons of proteins in the two groups were conducted, differentially expressed proteins associated with galectin-7 expression were identified and are listed in Table 3.

**TABLE 3:** Detection of galectin-7-related differentially expressed proteins through iTRAQ, gene ontology (GO), and KEGG analyses.

Description (Symbol)	Galectin-7 gene	Related proteins	Gal-p:WT	Gal-i: WT
Gal-p:WT	H. E	Calsequestrin	2.45	
		Keratin, type II cytoskeletal 4	2.21	
		Myosin light chain 1/3 -skeletal muscle isoform	2	
		Myosin-1	1.94	
		Myoglobin	2.10	
		Myosin light chain 1/3	2	
		Myosin regulatory light chain 2	2.34	
		Creatine kinase M-type		0.82
Gal-i: WT	L. E	Keratin, type II		0.78
		Keratin, type I cytoskeletal 13		0.53
		Myosin regulatory light chain 2		0.78
		Myosin light chain 1/3		1.94
		Myosin, heavy polypeptide 8		0.79
		Troponin C		0.70

*H.E, highly expressed; L.E, low expressed; Gal-p, galectin-7 transgenic; Gal-I, galectin-7 interfere; WT, wild type.*

Screening of the 1,009 identified differentially expressed proteins revealed that 28 of them were known proteins (Table 3). Notably, expression of some of these proteins relatively changed in knockdown mice expressing less galectin-7 as compared to WT mice and galectin-7-overexpressing mice, with levels of proteins “Serum amyloid A protein,” “Actin 1,” observed to be relatively increased in knockdown mice, whereas levels of proteins “Myosin regulatory light chain 2,” “Keratin (type I cytoskeletal 13),” “Troponin C,” “Creatine kinase M-type,” “Myosin light chain 2,” and “Myosin, heavy polypeptide 8” were relatively decreased in knockdown mice. By contrast, “Calsequestrin,” “Myosin light chain 1/3,” “Myoglobin,” “yosin regulatory light chain 2,” “Myosin-1,” and “Keratin (type II cytoskeletal 4)” were progressively increased in galectin-7-overexpressing mice. To further characterize both the total set of identified proteins and the subset of proteins with an altered expression between knockdown and galectin-7-expressing mice, biological function and pathway analyses were performed using the above-mentioned online resources. Ultimately, the results of these analyses revealed that the most highly enriched GO functional categories associated with differentially expressed proteins were associated with signal transduction and molecule metabolic processes.

### Intracerebral Hemorrhage Patient’s Characteristics

In this study, we studied the entire genomic DNA sequence and neighboring sequences of *LGALS7* genes of 24 patients with stroke and 70 healthy controls belonging to the Chinese Han population. Clinical characteristics of the 24 patients with stroke are shown in Table 1. The mean age of patients with stroke was greater than that of healthy controls. In addition, rates of hypertension, diabetes, and lipid disorders in cases were significantly greater than corresponding control rates. As expected, traditional vascular disease risk factors were more common in the stroke group (*p* < 0.001) than in the control group (healthy volunteers).

### Single-Nucleotide Polymorphism Genotyping Results

**FIGURE 4:**
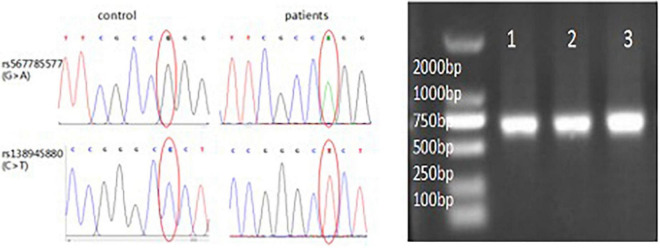
Homozygosity of *LGALS7* SNP alleles in patients with ICH. *LGALS7* gene sequencing electropherogram showing sequences in regions of SNPs rs567785577 and rs138945880. Right figure: results show successful insertion of promoter region DNA of the *LGALS7* gene into the vector (750 bp). Results of electrophoresis of PCR products revealed predicted results for the patients with ICH (lane 1) and two control healthy subjects (lanes 2–3), respectively.

Results that demonstrate significant contributions of *LGALS7* SNPs to ICH risk are shown in Table 4, whereby *LGALS7* SNPs rs567785577 (A) and rs138945880 (T) were each associated with greater risk of stroke (Figure 4). Testing to assess homozygous and heterozygous frequencies of alleles associated with both of these SNPs in the stroke cohort revealed that they were in HWE (*p* > 0.05). However, we failed to observe these alleles in homozygous form in individuals of the healthy volunteer group.

**TABLE 4:** Polymorphic analysis and statistical analysis of the promoter of galectin-7 genotypes.

Chromosome Location	dbSNP ID	Location	Gene	HWE *P*-value	*P*-value		Cases	Control	Odds rations	95% CI
19q13 GRCh38 GRCh37	rs567785577	38773638 39264278	LGALS7	0.9772	0.0623		24	16	2	0.9404–4.222
19q13 GRCh38 GRCh37	rs138945880	38773693 39264333	LGALS7	0.7715	0.045		24	21	1.643	0.8284–3.325

**Samples**	**Genotype of SNP**		**allele**	**Number of cases**		**Number of controls**			**Odds ratio (95%CI)**	***P-value***

Cerebral hemorrhage	TT		T	24,	48	16,		64	2 (0.940–4.222)	0.068
	AA		A	24,	48	21,		69	1.643 (0.940–4.222)	0.068
Controls	TC	AG	C	0,	0	32	27	62	0.533 (0.267–1.048)	0.086
	CC	GG	G	0,	0	15	15	57	0.6 (0.309–1.163)	0.169

*HWE, Hardy–Weinberg equilibrium; ORs, odds ratios; CI, confidence intervals.*

Hardy–Weinberg equilibrium of SNP alleles of controls was assessed using *Mnl*I or *Mbo*I digestion of SNP DNA. Genotyping of rs567785577 (c.146 + G > A) yielded three fragments (92, 15, and 5) for allele G and two fragments (122 and 102) for allele A when digested with *Mnl*I. Meanwhile, genotyping of rs138945880 (c.201 + C > T) yielded three fragments (88, 13, and 11) for allele C and two fragments (114 and110) for allele T when digested with *Mbo*I. Based on these results, frequencies of A and T polymorphisms of these SNPs in this Chinese Han population were determined to be 8.571% (95% CI:1.41–93.99, *p* = 0.031) and 13.5% (95% CI: 2,249–146.5, *p* = 0.002), respectively, for an overall frequency of polymorphic alleles of 10.7% (95% CI: 2.75–46.63, *p* < 0.0001).

### Association of Single-Nucleotide Polymorphism-Associated Allele and Genotype Frequencies With Intracerebral Hemorrhage Risk

Further analysis of haplotype structures of critical common *LGALS7* SNPs associated with cerebral hemorrhagic stroke revealed two haplotype blocks covering the entire study population. The polymorphic *LGALS7* gene existed as two major alleles, T and A, with the A allele occurring with greater frequency (T: *A* = 1:1.09). However, both alleles were found to be the important risk factors for ICH based on the following results: Allele T (OR = 13.5, 95% CI = 2.249–146.5, *p* = 0.002), Allele A (OR = 8.571, 95% CI = 1.41–93.99, *p* = 0.031).

## Discussion

Population-based genetic association studies provide more statistical power than family-based linkage studies for determining associations between oligogenic or polygenic genetic risk factors and human diseases. This study of a Chinese Han population was conducted to verify vascular disease-associated gene signals identified in the first phase of our study (the exploration phase). Our results revealed that two *LGALS7* intergenic SNPs, rs567785577 and rs138945880, were associated with non-traumatic cerebral hemorrhagic stroke. The association of these SNPs (within the 19q13.2 genomic region) with ICH was determined based on an independent dataset obtained from patients with ICH. To our knowledge, this is the first report of a positive association of rs567785577 and rs138945880 SNPs within the gene-encoding galectin-7 (*LGALS7*) with cerebral hemorrhage risk, although these results await validation through studies of larger cohorts of ICH cases and healthy controls. Notably, the atherosclerosis risk factor profile was found to be most significantly correlated with ICH stroke risk in this Chinese Han population cohort.

Different genetic profiles among individuals of different populations may be responsible for contradictory genotype and allele frequency results across studies, whereby a gene that is associated with disease susceptibility in one population may not be associated with susceptibility to the disease in another population (Sacco et al., 2006; Pantoni, 2010; Shao et al., 2019). Therefore, results that show significant associations between SNPs and a given disease should be extensively replicated and validated through studies of independent cohorts. In this study, we provide new evidence supporting an association between a common genetic variant and non-traumatic ICH risk. Meanwhile, other recent studies have identified additional candidate genes associated with ICH risk that include *SLC22A44* and *PMF1* loci. Collectively, all of these results together with the results of other studies indicate that genetic variation appears to influence ICH risk and disease characteristics. In addition, roles of genetic variants vary in importance depending on a patient’s age and also on frequencies of disease-associated variants in the general population, as reflected by HWE values (*p* > 0.05). Nevertheless, the growing list of gene loci associated with stroke risk highlights the fact that biological processes are associated with disease characteristics related to disease onset and progression. In this work, people of 50 years of age and older who carried certain *LGALS7* alleles were found to be at increased risk for ICH, especially those with hypertension and diabetes (*p* < 0⋅0001). Importantly, accelerated identification of susceptibility genes and biological pathways has transformed our understanding of disease associations with complex traits (Falcone et al., 2014) by revealing that the risk of contracting a given disease can vary greatly depending on whether a person possesses one vs. multiple disease susceptibility alleles, in addition to increased risks associated with aging.

It has become increasingly clear that aging has diverse effects on different tissues involving alterations of gene expression patterns and physiological processes (Jonker et al., 2013). For example, changes in the expression of inflammatory and stress response genes have been shown to increase with advancing age, which indicates that at the gene expression level, normal aging is a slowly progressive process (Srivastava et al., 2020). Moreover, changes in the expression of genes encoding brain proteins (e.g., glial fibrillary acidic protein), genes within the complement pathway, and those associated with immune system functions appear to be associated with age-related inflammation.

In this work, galectin-7 was found to be associated with the expression of numerous brain proteins as a possible mechanism by which *LGALS7* expression influences ICH risk. For example, galectin-7*-*associated cerebrovascular wall-related proteins and differentially expressed proteins were found to be significantly correlated with *LGALS7* expression in TG mice, as observed in several previously reported studies (Al-Ansari et al., 2009). In turn, cerebrovascular accumulation of β-amyloid (Aβ) has been shown to disrupt blood flow within the brain that adversely affects both the vascular blood supply and functions of astrocytes, vascular endothelium, and numerous associated nutrient transporters (e.g., glucose transporter GLUT1 and galectin-7-associated monocarboxylate transporter MCT1) (Pierre et al., 2000; Merlini et al., 2011). Moreover, the activity of yet another galectin-7-associated protein, internal membrane protein alpha-1-acid glycoprotein 2, can modulate blood-brain barrier permeability to charged molecules by adding negative charges to the matrix component of the blood-brain barrier (Yuan et al., 2010) that influence functions of synthetic and contractile smooth muscle cells (SMCs) located within the middle layer of cerebral vessel walls (Poittevin et al., 2014). These functions depend on α-actin expression, as supported by an *in vitro* study of rat SMCs that expressed α-actin and exhibited contractile phenotypes as determined *via* immunocytochemical analysis (Quelhas et al., 2020). Meanwhile, another galectin-7-associated protein, myoglobin, regulates nitric oxygen generation and activity within cerebral blood vessels, whereby when the regulatory effect of myoglobin was weakened due to silencing of galectin-7 expression, the concentration and function of nitric oxide will be affected. Another brain protein, serum amyloid A (SAA), is involved in the chemotactic recruitment of inflammatory cells to the site of inflammation (Cunnane and Whitehead, 1999). Intriguingly, silencing of *LGALS7* that was conducted in this study to assess differential protein expression led to a progressive increase in SAA levels in mice after the knockdown. Taken together, the results described above indicate that silencing of galectin-7 expression may inhibit actin-driven angiogenesis by suppressing actin-driven endothelial cell activities, actin contractility, and cell migration properties that triggered the development of cerebral amyloid angiopathy (CAA) in these mice. CAA predominantly affected both perivascular and transmural inflammatory processes that supported extensive immunoreactivity around blood vessels and triggered angiitis, a related angio-destructive process that led to thickening of vascular basement membranes (VBMs) and SMC degeneration (Frackowiak et al., 1994; Kozberg et al., 2021). Notably, the recurrence rate of CAA in one group of patients receiving immunosuppressant treatment was 26% as compared to 71% in those not receiving immunosuppressant treatment (Moussaddy et al., 2015).

In this study, an iTRAQ quantitative proteomic analysis was conducted that revealed associations between differentially expressed proteins belonging to unique signaling pathways and characteristic proteins involved in ICH. Our results confirmed that changes in galectin-7 expression in middle-aged, diabetic, hypertensive, and dyslipidemic patients were associated with changes in levels of downstream troponin proteins as a possible mechanism to explain increases of serum troponin levels in patients with increased arterial stiffness or hemodynamic abnormalities. However, the value of conducting routine surveillance of troponin levels to provide independent estimates of long-term postoperative outcomes is uncertain (Filipovic et al., 2005). Nevertheless, whether triggered by subarachnoid hemorrhage or intracranial hematoma, increased troponin levels in the body can lead to strong systemic stress responses and enhanced release of catecholamines that can result in neurocardiac imbalance (Naredi et al., 2000; Wenzl et al., 2006; Sykora et al., 2012). Therefore, it has been suggested that galectin-7 can influence the expression of myosin and actin proteins in vascular intima and vascular smooth muscle by influencing signaling pathways involving signaling molecules such as kinase C (PKC), Rho kinase, G protein, and oxidative stress. Meanwhile, galectin-7 also influences inflammatory signaling pathways by interacting with myosin light chain 1/3, myosin-1, myosin, myoglobin, and troponin C proteins (Smeda et al., 1999; Shimomura et al., 2004; Khan et al., 2006; Hayakawa et al., 2011; Ghiso et al., 2014; Zhou et al., 2018; Xue-Qin et al., 2020; Take et al., 2022). Taken together, the results of this study and numerous other studies indicate that the galectin-7 is involved in the regulation of activities of a relatively large number of related proteins in diverse pathways and proteins common to multiple signaling pathways.

Our study had a number of limitations, including a sample size that was too small for determining associations between SNPs and ICH risk in a population with Han ancestry. Studies conducted based on larger sample sizes will be crucial to validate our findings. Furthermore, only a small number of patients with ICH stroke were included in this study as compared to a number of control subjects (healthy volunteers). In addition, associations between *LGALS7* SNPs with subarachnoid hemorrhage and ischemic stroke were inconclusive and thus were not included in the results of this study. Finally, there was a possibility of false positives, owing to differences in the age distributions between cases and controls, with the older age of the study group correlating with greater ICH risk.

## Conclusion

The results of this study support the role of genetic factors in human ICH, while also providing new insights into ICH pathogenesis that will likely broaden the scope of therapeutic interventions for preventing and treating this common disabling condition.

## Data Availability Statement

The data presented in the study are deposited in the GenBank repository https://www.ncbi.nlm.nih.gov/nuccore/OM743281, accession numbers OM743281–OM743304.

## Ethics Statement

The studies involving human participants were reviewed and approved by the Ethics Committee of The First Affiliated Hospital of Xiamen University. The patients/participants provided their written informed consent to participate in this study.

## Author Contributions

M-DW and M-JS: conception and design. M-DW, M-JS, JT, and Z-XW: acquisition of data. M-DW, JT, and M-JS: analysis and interpretation of data. M-DW, M-JS, and JZ: drafting the article. Z-XW, S-YZ, and M-JS: critical revision of the article. All authors: reviewing the submitted version of the manuscript. S-YZ, M-JS, and Z-XW: approval of the final version of the manuscript on behalf of all authors. M-DW, M-JS, and JT: statistical analysis. S-YZ, M-JS, and Z-XW: administrative/technical/material support. S-YZ, M-JS, JZ, and Z-XW: study supervision. All authors contributed to the article and approved the submitted version.

## Conflict of Interest

The authors declare that the research was conducted in the absence of any commercial or financial relationships that could be construed as a potential conflict of interest. The reviewer YC declared a shared parent affiliation with the authors JT and S-YZ at the time of review.

## Publisher’s Note

All claims expressed in this article are solely those of the authors and do not necessarily represent those of their affiliated organizations, or those of the publisher, the editors and the reviewers. Any product that may be evaluated in this article, or claim that may be made by its manufacturer, is not guaranteed or endorsed by the publisher.

## Footnotes

1
https://www.genome.jp/kegg

2
https://string.embl.de/
