# Supplementary material for: Human Galectin-7 Gene LGALS7 Promoter Sequence Polymorphisms and Risk of Spontaneous Intracerebral Hemorrhage: A Prospective Study
Source: Front Mol Neurosci. 2022 Mar 23;15:840340. doi: 10.3389/fnmol.2022.840340 (PMC8984465; doi:10.3389/fnmol.2022.840340)
Supplement: Supplementary file 1 [file Table_1.docx]

**Human Galectin-7 Gene *LGALS7* Promoter Sequence Polymorphisms and Risk of Spontaneous Intracerebral Hemorrhage: A Prospective Study**

*Wang, et al*

Supplementary Appendix-total-1

**Section page**

**1) LGALS7 promoter and shRNA sequence selection and Design 1**

**2) Vector Construction 2**

**3) Animal assays (Transgenic mice, Wild-type BALB/c mice, Interfere with mice) 6 4) Isobaric tags for relative and absolute quantification(iTRAQ) 7**

**5) Western blotting 8**

**6) Locus Mapping 9**

**Section 1. LGALS 7 promoter and shRNA sequence selection and Design**

DNA sequences and amino acid sequences were analyzed using BLAST (<http://blast.ncbi>. nlm.nig.gov/Blast.chi), GenBank database. Promoter prediction: find genomic DNA sequence relative to the upstream of the Galectin-7 initiation code, A 6-10kb segment was used for promoter analysis to estimate the starting site of the promoter. Using the following website prediction, a common prediction region was selected to design PCR primers. using the Promoter Scan (http://wwwbimas.cit.nih.gov/molbio/proscan/), Promoter 2.0 Prediction Server (http://www.cbs.dtu.dk/services/Promoter/) and Berkeley (<http://www.fruitfly.org/seq_tools/> promoter.html).

Open reading frames (ORFs) were predicted using the ORFs finder (<http://www.ncbi.nlm>. nih.gov/orffinder). Sequence from the ORF gene of LGALS 7 were designed by siDirect and manual selection and based on the website siRNA designing tools (<http://design.RNAi.jp/>, Ambion software design the sequence of interference hairpins, Insert Design Tool for the pSilencer™ Vectors). 23-base candidate target sequence and the online mouse genome were by BLAST to ensure the sequence without a perfect match is selected as the Gelectin-7 gene interference target sequence.

The non-specific sequences provided by psilencer5.1 vector kit(Ambion USA), which had no significant nucleotide sequence homology with human, rat and mouse genomic DNA, were used as negative control sequences. Two theoretically valid sequences 143 to 161and 265 to 283 were selected. Nonspecific sequences were also scanned by BLAST analysis and served as negative control and was named as negative -shRNA. All the sequence were arranged in the following alignment: Bbsl+ Sense +loop +Antisense +Termination signal +BamH І+ Hind Ⅲ and cloned into the linearization pSilencer 5.1 vector with BamH I and Hind III double enzymes to make shRNA expressing plasmids: pSilencer-galectin-7- shRNA1, pSilencer-galectin-7- shRNA2, and pSilencer-negative- shRNA.

The designed interference hairpin sequence is as follows：The shRNA template [ shRNA1-Forward strand: 5'-GATCCGGGCACTGTCATGAG AATTCTCAAGAGAAATTCTC ATGACAGTGCCCACTTTTTTGGAAA-3';

Reverse strand:5'-AGCTTTTCCAAAAAAGTGGGCACTGTCATGAGAATTTCT CTTGAGAATTCTCATGACAGTGCCCG-3';

shRNA2-

Forward strand: 5'-GATCCGCTTCCGAGGTTGTCTTCAATTCAAGAGATTGA AGACAA CCTCGGAAGTGTTTTTTGGAAA-3'; Reverse strand: 5'-AGCTTTTCCAAAAAACACTTCCGAGGTTGTCTTCAATCTCTTGAATTGAAGACAACCTCGGAAGCG-3'].

**Section 2.**  **Vector Construction**

**Construction of pcDNA3.1-galectin-7- plasmid** **Vectors**

The galectin-7 promoter regions(639bp) were amplified by polymerase chain reaction (PCR) using designed DNA served as templated as the template and the following primers1 and 2: upstream primer 1: 5ʹ-TCAGGTACC/AGTCCAGGCAGGAGGTTC-3ʹ (primer 2: 5ʹ-TCAGGTACC/AGTCCAGGCAGG AGGTTC-3ʹ) and downstream primer 1:5ʹ-AAGCTCGAG/TGAGACCGAAGGAGGGAG-3ʹ (primer 2: 5ʹ-AAGCTCGAG/GAGGAAGCCACAAAGAGGTAGAGGG-3ʹ). The 639bp fragment of Galectin-7 gene promoter region was amplified by PCR. Promoter DNA was separated using electrophoresis on agarose gels (1%, w/v). A band corresponding to galectin-7 was excised and the DNA was extraction using a gel Extraction kit (TIAN gel Midi Purification Kit). The two fragments obtained were ligated with T4DNA ligase at 16°C, and 10μl of the ligation products were taken and transformed into competent cells. After transfected, positive clones were selected, extract, purify and then double digestion with Kpn I and XhoI. To identify whether the target gene is inserted into the promoter region of the reporter plasmid pGL3-basic, which inserts a specific fragment of Galectin-7 promoter (639bp) into the front of the luciferase expression sequence. Luciferase activity measurement, using Dual-Luciferase Reporter Assay System were performed according to the manufacturer’s instructions (Promega, USA), repeated 3 times. The recombinant strains were selected on LB agar plates containing ampicillin. The recombinant plasmid (pGL-3-basic, p-CGC-Luc,p-TGC-Luc, p-CAC-Luc,p-CGT-Luc, p-TAT-Luc) was isolated from E.coli transformant. Using LIP2000, 293T cells were transfected to detect promoter function.

Primer 5.0 software was used to design primers according to the PCR primer design principle and the galectin-7 gene sequence in GenBank. The upstream primer of the galectin-7 promoter was added to the Kpn I restriction site (GGTACC), the downstream primer was added to the Xho I restriction site (CTCGAG), and the protective base was added to the 5th end of the upstream and downstream primers, respectively. The primers were synthesized by Shenggong Co., Ltd. The length of the amplified fragment of the promoter was 639 bp. The amplification reaction conditions were: pre-denaturation at 95°C for 5 min; denaturation at 95°C for 1 min, annealing at 55°C for 30 s, extension at 72°C, 1 min, 35 cycles; 72°C extension for 10 min; 4°C Hold. The PCR product was sent to Shanghai Shenggong Biological Co., Ltd. for sequencing, and the sequence results were analyzed.

The PCR product was purified using a common agarose gel DNA recovery kit, and purified and confirmed by agarose gel electrophoresis. After confirmation, the PCR purified product was ligated to T-Vector pMD19 (Smiple). The ligation system was 1uL of T-Vector pMD19 (Smiple) vector, 1 uL of T4 DNA ligase, 1 uL of 10×T4 DNA ligase buffer, 7uL of PCR purification product, and the system was placed in a metal bath at 16°C for 6 h. The ligation product was transformed into E. coli JM109 competent cells. The positive colonies were inoculated into 10 mL LB medium containing ampicillin, and cultured at 37°C, shaking at 200 r/min overnight, and then identified by PCR, and recombinants (T-T and T-A) were obtained, respectively, and the plasmids were extracted. The plasmids T-T, T-A and pGL3-basic were digested with Kpn I and Xho I, respectively, and the purified products T and A were respectively ligated with the pGL3-basic vector, and the ligated product was transformed, and the plasmid was extracted and digested. By sequencing, the recombinant vectors were named pGL3-T and pGL3-A.


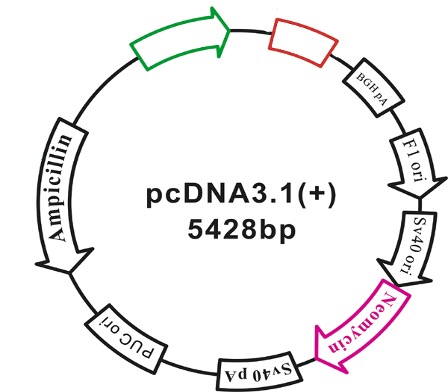


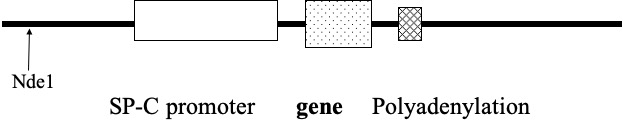


**Supplementary Figure 1**. Plasmid structure diagram of an overexpressed promoter

**Construction of pcDNA-galectin-7-shRNA+ pSilencer-galectin-7-recombinant plasmid** **Vectors, pcDNA3.1(+) (Invitrogen, USA)**

To construct the expression vector of shRNA that can specifically inhibit the galectin-7 gene expression, we selected specific sequence according to the sequence of mouse mRNA, and then designed pairs of oligos for hairpin RNA expression, can chemically synthesized and annealed.

The full length of OLC1 was released from the eukaryotic expression vector of OLC1-PCDB using EcoR1 endonuclease and inserted into the EcoR1 site of the 3.7SPC/SV40 vector, and then modified vector 3.7SPC/SV40 was linearized and annealed inserted into the downstream of the pcDNA3.1 vector’s SPC promoter to construct recombinant RNAi plasmids. Competent Escherichia coli cell were transfected by pcDNA3.1-galectin-7-shRNA, and then extracted from the transfected E.coli for sequencing performed.

Linearized transgenic vectors were injected into the fertilized eggs of synchronously treated donor female mice by microinjection. Pseudo-pregnant recipient female mice (produced by mating between normal female mice and ligated male mice) were used to conceive the fertilized eggs that survived the injection, and finally the F0 generation transgenic mice were produced.

Galectin-7 interferes with the validation of mouse models (Plasmid stability analysis): the first to establishment interfered with mouse PCR detection (Fo-F3 generation, upstream, downstream primer see Table1).


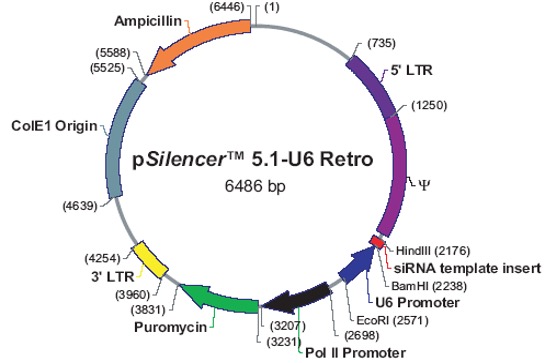


**Supplementary Figure 2.** Plasmid structure diagram of silent expression

**The control of plasmid transfection efficiency, pSilencer 5.1 (Ambion USA)**

We have found the influence of the difference in transfection efficiency on the fluorescence in the preliminary experiments, so we directly used the dual-luciferase reporter gene detection system kit from American Promega Company to overcome this difference. The Renilla fluorescence in this system is sufficient to offset the effect of different transfection efficiencies and cell numbers on fluorescence. The firefly fluorescence used in this dual fluorescence detection system is not endogenously expressed in mammals and is not affected by other components in cells. The vector carrying the promoter of the gene of interest in this study,

After co-transfection with a dual-luciferase reporter gene; Using instruments to detect both types of fluorescence. The ratio of firefly fluorescence to that of Renilla fluorescence was plotted as a histogram. Eliminates differences in cell number and transfection efficiency even with different transfection efficiencies.

**Section 3.**  **Animal assays (Transgenic mice, Wild-type BALB/c mice, Interfere with mice)**

**Cell Culture and Luciferase Reporter Gene Assays**

Human embryonic kidney cells 293T were cultured in a high glucose type DMEM medium containing 10% fetal bovine serum at 37°C, 5% CO_2_, and a relative humidity of 95%. Digestion was performed once every 2-3 d with 0.1% trypsin. One day before transfection, 293T cells were inoculated into 48-well plates. When the cell growth reached 70%-80%, two experimental groups and one control group were set up, and three replicates were set in each group. Dilute with 25 uL of serum-free diluent and mix well to prepare DNA dilution. Add Lip2000 Neofect TM 1ul, dilute with 25 uL of serum-free dilution, mix gently, let stand for 5 min at room temperature, then dilute the DNA and transfection solution. Mix and prepare the transfection complex, let stand for 20 min at room temperature, add the transfection complex to the cell culture, mix gently, and change the solution after 4-6 h.

After transfection for 48 h, the culture solution was discarded, and the cells were washed with PBS, and 65 uL of PLB (Passive lysis buffer) was added to each well, and the cells were fully lysed by shaking at room temperature for 15 min. Using a multi-function microplate reader, 20uL of the product was added to a white opaque 96-well plate, 100 uL of LARII working solution was added to each well, and mixed for 2 s for 5 s. After the reading is completed, add 100 uL Stop & Glo® Reagent to each well, mix for 2 s, and read for 5 s; determine the firefly luciferase activity and the internal renilla luciferase activity, and calculate the ratio between the two. The basis for measuring the activity of the promoter.

**Section 4.**  **Isobaric tags for relative and absolute quantification(iTRAQ)**

iTRAQ combined with liquid chromatography-tandem mass spectrometry (LC-MS/MS) analysis has become a powerful quantitative proteomic approach, it has significant advantages of high throughput, high sensitivity and higher accuracy.

**iTRAQ sample preparation, protein extraction and iTRAQ labeling**

In this study, rats were separated: wild type (BALB/c) group, LGALS7 transgenic mice group, LGALS7 Interference mice group, three groups of 1-month-old mice with 2 mice in each group were selected for tail positive identification and protein extraction. Blood and lung were collected, an appropriate amount of cracking buffer (7M urea, 2M thiourea, 0.1% Chaps) was added to the tissue sample and then mixed by vortex. Ultrasound was on for 60 s, on for 0.2 s, off for 2 s, and the amplitude was 22%. tissue sample at 1500g for 20min at 4 °C, the whole blood at 1500g for 10min at 4 °C, and stored at -80°C for proteomic analysis.

Proteins(200μg) were incorporated into 4μL Reducing Reagent for 1 hour at 60°C, add 2μL Cysteine-Blocking Reagent, at 12,000g for 20min. then added 100μL dissolution buffer three time were washed. Finally, the protein suspensions were digested with 4μg trypsin in 50μL DS buffer overnight at 37°C, and the resulting peptides were collected as a filtrate. Peptide mixture(100μg) was labeled using iTRAQ regent according to the manufacturer’s instructions, then, the collected fraction were desalted and concentrated by centrifugation, and lyophilized. The labeled peptide mixture were dissolved in 100μL mobile phase A, centrifuged at 14,000g for 20min, and supernatant was taken for use. The separation was performed with 400μg enzymolysis BSA. Take 100μL of the prepared sample, at a flow rate of 0.7ml/min, a liner gradient program was shown as followed: 5-18%buffer B for 40min, 32-95%buffer B for 138min, 95-5%buffer B for 100min.

The peptide mixture obtained by high pH reversed phase separation, draw supernatant and load the sample. The Loading Pump at a flow rate of 350nL/min for 15min. the separation gradient was shown as followed: 4-15%buffer B for: 5min, 25-35% buffer B for: 105min, 95% buffer B for: 70min, hold 95%buffer B for: 82min, 4%buffer B for: 85min, hold 4%buffer B for 90min.

The database used this time is the UNIPROT Mus musculus database. The iTRAQ mass spectrometric analysis was performed using the Thermo Q-HF-X mass spectrometer, and the original mass spectrometric file was processed by Proteome Discoverer1.3, a commercial software supported by Thermo. After filtering according to the ratio of the original data, the difference multiple was ≥1.2 or ≤0.833, and the obtained results were entered into the analysis.

**Section 5.**  **Protein extraction, Western blotting (WB)**

Protein extraction was performed by incubating for 30 min on ice and subsequently centrifuging for 30 min at 4 °C and 13,000 rpm. Supernatants were quantified using the Bio-Rad Protein Assay Dye Reagent Concentrate (Bio-Rad, Munich). 80μg protein/lane was used for Western blotting. After transfer, the PDVF filters were incubated with the following antibodies: anti-human Galectin-7, anti-Tubulin (G8), sc-55,529(Santa Cruz Biotechnology, USA). Blots were developed using the Western lightning plus-ECL system (Perkin Elmer, Rodgau).

Cells and tissue After the mice tissues were weighed, they were placed in a mortar and mortar, and tissue/cell lysate buffer was added to grind at a ratio of 0.5mg/500ml, and the tissues/cell were transferred to a 1.5ml centrifuge tube. Centrifuge at 12000g at 4℃, centrifuge for 5min, boil the sample in boiling water for 5min, denature the protein, place it on ice, Supernatants were electrophoresed on 10% SDS-PAGE, and transferred to polyvinylidene diuoride (PVDF) membranes, which were then blocked with 5% non-fat milk. Immunoblot analysis was undertaken by incubation with primary antibodies (1:1000) at 4 °C overnight. After washing, membranes were incubated for 1 h at room temperature with horseradish peroxidase-conjugated goat anti-mice IgG (1:4000).

**Section 6.**  Locus Mapping

639bp promoter sequence

AGTCCAGGCAGGAGGTTCTCAGCTGGAGAGAAGGGTCTGGAGTTCCCACCCAGATTGGCCTCCCAGGACCTAATATGTCTGTGTATAAAATAAGAGAAATTACTAACAAATAAATACATGAAATGAGATAGAGGCTCTCCGTGTGCCAGGCAGCGGGAAGACAGGGGTGGAGGGGCAGTGGCACAAGGAGCCTTCTCTGCTGTGAGTTTATTACTATTAAACATTCCCTCTTATTATCAGCTCCTAGGAGAGGGGCAGTGCACCCGGGCCCTGGGTGATGGGGGGATCAGGGCCCCCAAGAAGAGGTGCCACTCCCGCTTCGCCGGGCTTGCCCCAGGGCCAGGGTCCGTGCCAGGTGTGGGTGGTGGGTGGGAAGGGGTGGGGTGAGTCATCAGGGCCAGCCCCGCCCTGCTTTTATTTAAGGTCCCCAGCAGGCCCCACCACC**A**CGGCTGCCCAACCCGGTCCCAGCCATGTCCGTGAGTGCTCCAGGGGCCCGGGGCGGGGCCAAGCAGGGAGGGGGCTGGGGGCTCCTCTTTCAAAGGAGCAAGTGGGCAGTGAGTGTGCCACGGACTGGCCTGGGCTGTGGCCTCCGGGTAACCCCCATCCCGACTGTCCCCTCTACCTCTTTGTGGCTTCCTC

[rs567785577](https://www.ncbi.nlm.nih.gov/projects/SNP/snp_ref.cgi?rs=567785577)：

ACGGACCCTGGCCCTGGGGCAAGCC[C/T]GGCGAAGCGGGAGTGGCACCTCTTC

Antisense strand：GAAGAGGTGCCACTCAGGCTTGCCCCAGGGCCAGGGTCCGT

[rs138945880](https://www.ncbi.nlm.nih.gov/projects/SNP/snp_ref.cgi?rs=138945880)：

GGGCCCTGATCCCCCCATCACCCAG[A/G]GCCCGGGTGCACTGCCCCTCTCCTA

Antisense strand： TAGGAGAGGGGCAGTGCACCCGGGC T CTGGGTGATG GGG GGATCAGGGCCC

RS567785577：Before ATG-146

RS138945880：Before ATG-201

**GenBank: OM743281-OM743304**
